# Prone positioning combined with high-flow nasal or conventional oxygen therapy in severe Covid-19 patients

**DOI:** 10.1186/s13054-020-03001-6

**Published:** 2020-05-26

**Authors:** Cyrielle Despres, Yannick Brunin, Francis Berthier, Sebastien Pili-Floury, Guillaume Besch

**Affiliations:** 1 Department of Anesthesiology and Intensive Care Medicine, University Hospital of Besancon, University of Franche-Comte, Besancon, France; 2 EA 3920, University of Franche-Comte, 3 bvd Alexander Fleming, 25000 Besancon, France

Dear Editor,

A massive outbreak of coronavirus disease 2019 (Covid-19) occurred in France in March and April 2020. About 20% of Covid-19 patients develop acute respiratory distress syndrome (ARDS), with mortality ranging from 20 to 50%. Since the publication of the PROSEVA study [1], prone positioning (PP) has become a cornerstone of management of mechanically ventilated severe ARDS patients.

Recently, PP was reported to enhance oxygenation when combined with high-flow nasal cannula in severe non-Covid-19 ARDS [2, 3] and to improve lung recruitability when combined with non-invasive ventilation in severe Covid-19 ARDS [4].

We report the case of 6 severe Covid-19 patients admitted to our critical care unit between March and April 2020, who had PP combined with either high-flow nasal oxygen (HFNO) or conventional oxygen therapy (COT). All patients had laboratory-confirmed SARS-CoV-2 infection, defined as a positive result of real-time reverse transcriptase-polymerase chain reaction (RT-PCT) from nasal and pharyngeal swabs. ARDS was defined according to the Berlin definition, with a ratio of P_a_O_2_ to F_i_O_2_ (P_a_O_2_/F_i_O_2_) ≤ 300 mmHg. All patients presented rapid worsening of dyspnea and oxygenation, defined as S_p_O_2_ ≤ 92% despite increasing oxygen supply to more than ≥ 5 L/min. All patients were spontaneously ventilated, and no patient had criteria that indicated the need for emergency intubation. All patients had predominant posterior lung condensation documented either on lung ultrasound or CT-scan.

HFNO or COT was prescribed to reach S_p_O_2_ ≥ 94%. The clinical course of ARDS was closely followed using the ROX index [5]. PP was proposed to patients who presented clinical worsening, as persistent hypoxia despite increasing oxygen delivery, or a decrease in the ROX index. PP was maintained depending on patient clinical tolerance and could be repeated if necessary.

Relevant clinical, laboratory data and HFNO or COT settings were obtained from medical records and are presented in Table 1.

**Table 1:** Clinical characteristics and outcomes of patients

Case no.	Gender	Age (years)	SAPS II score at admission	Ventilatory support	BMI (kg.m^−2^)	Duration of prone positioning (hours)	P_a_O_2_/F_i_O_2_ before prone position	P_a_O_2_/F_i_O_2_ after prone position	Intubation
1	Male	60	27	HFNO 50 L/min	27	7	144	254	Yes
2	Male	54	32	COT 6 L/min	27	1	215	147	No
				HFNO 50 L/min		1	129	156	
3	Male	55	26	HFNO 50 L/min	26	16	126	194	No
				HFNO 50 L/min		16	183	162	
4	Male	66	37	COT 5 L/min	31	4	150	242	Yes
5	Male	61	28	COT 3 L/min	21	1	274	225	Yes
				COT 3 L/min		2	193	124	
6	Male	64	36	COT 5 L/min	27	2	212	168	No

F_i_O_2_ with COT was calculated using the following formula: F_i_O_2_ = 21 + (4 × oxygen flow rate in L min^−1^)

*BMI* body mass index, *HFNO* high-flow nasal oxygen, *COT* conventional oxygen therapy

A total of 9 PP sessions was performed in 6 patients. PP was combined with HFNO in 4 sessions and to COT in 5 sessions. The P_a_O_2_/F_i_O_2_ ratio improved after 4 sessions, including 3 sessions combined with HFNO and 1 session combined with COT. Intubation was avoided in 3 patients.

This is the first report of PP combined with either HFNO or COT in severe Covid-19 pneumonia. The proportion of patients with P_a_O_2_/F_i_O_2_ ratio improvement after PP appeared to be higher with HFNO compared to conventional oxygen therapy, suggesting the need for a high flow of oxygen to provide a significant oxygen response [6]. All patients described subjective enhancement of dyspnea after prone positioning, but this data was not quantified. The efficacy of PP combined with HFNO therapy or non-invasive ventilation was recently reported in small cohorts of non-infectious and infectious non-Covid-19 ARDS patients [2, 3]. Interestingly, the proportion of patients with an improvement in P_a_O_2_/F_i_O_2_ ratio and the rate of intubation avoided in these 2 studies were very close to that observed in the present series of 6 severe Covid-19 patients.

Considering these observations, PP combined with either HFNO or COT could be proposed in spontaneously breathing, severe Covid-19 patients to avoid intubation. The indication for PP in non-intubated Covid-19 pneumonia needs to be addressed in further studies.

## Data Availability

Not applicable.
